# A Self-Powered Triboelectric Nanogenerator Based on Intelligent Interactive System for Police Shooting Training Monitoring and Virtual Reality Interaction

**DOI:** 10.3390/ma15186228

**Published:** 2022-09-08

**Authors:** Songyang Li, Changjun Jia, Fengxin Sun, Yongsheng Zhu

**Affiliations:** 1 Police Skills and Tactics Training Department, Criminal Investigation Police University of China, Shenyang 110035, China; 2 Physical Education Department, Northeastern University, Shenyang 110819, China

**Keywords:** triboelectric, intelligent system, self-powered

## Abstract

A self-powered triboelectric nanogenerator (SPTENG) based on triboelectric effect and an intelligent interactive system are fabricated for monitoring shooting training and virtual training. The SPTENG is composed of latex and PTFE and an intelligent system. Based on triboelectric effect, the SPTENG can be used to monitor the progress of trigger pressing without a power supply (this is supplied by trigger movements). Because of the flexible properties, it can be attached to a trigger conveniently to monitor the progress of trigger pressing, such as trigger time, trigger stability, etc. Meanwhile, as part of an intelligent shooting system, police can formulate a standard scheme according to signals to improve their skills. Furthermore, they can use it to train between reality and virtuality. Therefore, it has a wide development space in human–computer interaction and real-time information processing.

## 1. Introduction

With the development of fifth generation communication technologies for the Internet of Things [1,2,3,4], wearable devices are becoming increasingly common in the field of human health and human–computer interaction [5,6,7,8,9]. Wearable devices are able to be worn directly on the surface of the human body and are capable of sensing information about the human body. At the same time, they offer a wide range of applications, including motion monitoring [10,11,12,13,14,15], rehabilitation [16,17,18,19], human–machine interface (HMI) [20,21,22,23], disease diagnosis [24,25,26], etc., to further enhance human lifestyles. Among epidermal electronics based on biocompatible materials, an important class of wearable electronics, there has been significant development of skin-like biochemical monitoring patches [27,28,29], physical signal sensing systems [30,31,32,33], and miniaturized electronic incorporation into epidermal arrays. In addition, much work has been carried out using screen printing [34], inkjet printing [35], functionalization, and other techniques [36,37] to develop textile-based direct adhesion of wearable electronics [38]. These are widely used in healthcare, smart homes, and human–machine interfaces (HMIs).

There is little monitoring of the trigger for the police shooting system. In the shooting process, different people cannot pull the trigger completely with the same strength and direction due to different muscle types, palm size, finger length, training level, and gun holding methods. For example, the 92-type pistol, 77-type pistol, 64-type pistol, police revolver pistol, and 88-type sniper rifle are all used as live ammunition testing tools and the different weapons have different trigger force and trigger stroke. In the shooting process, the finger that pulls the trigger needs to be closely attached to the trigger, and the second joint of the index finger is used as the axis for bending and stretching. At the same time, other joints of the palm cooperate with it to form a joint force to complete the action of pulling the trigger. With or without support, the physiological functions before and after the movement are different. The traditional shooting training methods include empty gun preview, live-fire shooting, shooting control and monitoring system assistance, laser sight and electronic target machine analysis of impact point, etc. The above training methods can improve the shooting level to a certain extent, but the motion of firing fingers in shooting elements, especially the fitting degree between fingers and trigger before shooting, the direction and magnitude of exertion, and the exertion of palms, other fingers and auxiliary hands, cannot be monitored and analyzed. For example, different shooting habits can be distinguished, and very subtle differences can be detected. However, cameras and other equipment cannot capture them, and multiple devices need to work continuously, so multiple units cannot work at the same time, and the power supply of the equipment cannot be guaranteed.

In the firing process, the shooter’s finger micro-motion feeling relies entirely on the shooter’s perception and muscle memory brought by long-term training, which lacks effective monitoring means. The triboelectric nanogenerator (TENG) can be used as a sensing unit to acquire energy and sense information about the human body [32,39,40,41,42]. It has broad application prospects in the sensing field and has excellent sensing performance. It can monitor the movement track of the trigger finger, other fingers, and palm muscles during firing; monitor the fitting degree and force direction of the index finger and trigger; collect and analyze sweat when the hand grasps the gun; and provide various biomechanical and biochemical data for quantitative analysis, so as to give scientific guidance.

Based on friction electrification and electrostatic induction, this project studies a self-powered intelligent shooting system made of electronic skin to monitor physiological and biochemical indexes such as hand muscle movement track, force direction, and joint angle change during the shooting process of police officers, and to form feedback of the collected action information. The SPTENG is composed of latex and PTFE, and it can convert mechanical energy into electric energy. The electric energy not only is the trigger sensing signal but also the virtual interactive sensing signal. The whole process does not need an external power supply. Meanwhile, the SPTENG is the sensing unit in the intelligent shooting system; when the SPTENG generates a sensing signal, the shooting system will generate a feedback reaction. The self-powered intelligent shooting monitors the nuances of the shooting process to improve the firing action, thus improving the performance efficiently and providing a new path for the application of new materials.

## 2. Materials and Methods

### 2.1. Materials

The PTFE films (0.03 mm) and latex solution were bought from Taobao. The pistols and rifle belong to the National Police University of China.

### 2.2. Methods

First, the copper conductive wire was attached to the finger; then the latex solution was ejected onto the finger. The finger was in the still state for 10 min until the latex dried. The PTFE film, with a thickness of 0.03 mm, was attached to the copper foil and they were cut into pieces shaped like a trigger. Finally, the PTFE film with copper foil was attached to the trigger.

### 2.3. Test

The data for the properties and applications testing of TENG were tested by an oscilloscope (sto1102c, micsig which was produced by Shenzhen, China). The properties’ parts were tested by step motor. In practical testing, the SPTENG was attached to the trigger and to the finger, which were prepared for the performance test. For satisfying the requirements of wireless signal transport, we designed a wireless signal collection module. By integrating with this wireless signal collection module, the signal can be transported into the human–computer interaction system.

## 3. Results

**Figure 1:**
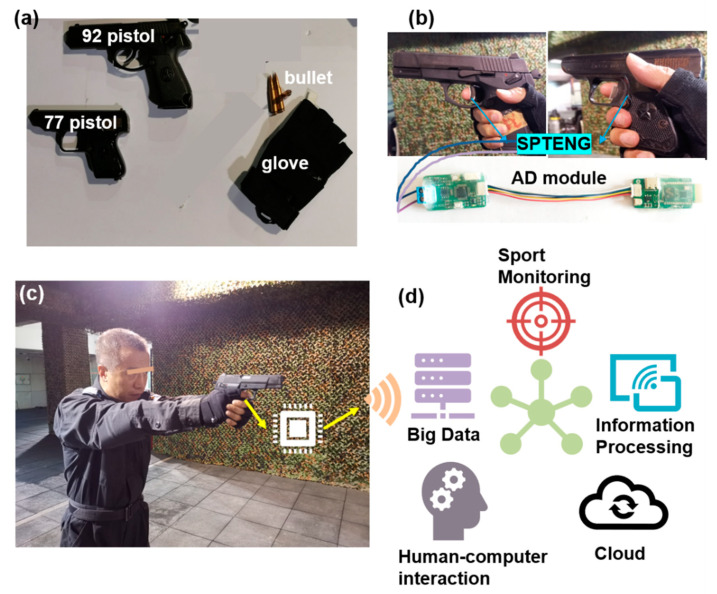
Self-powered intelligent shooting system for improving police shooting technology. (**a**) The image of a 92-type pistol, a 77-type pistol, bullets of 92-type pistol and 77-type pistol, and a sensor; (**b**) Pictures of testers holding a 92-type pistol and a 77-type pistol, and of an AD module; (**c**) Tester testing a 92-type pistol with sensors; (**d**) Intelligent shooting system application scene.

Images of a 92-type pistol, a 77-type pistol, thebullets of a 92-type pistol and a 77-type pistol, and a sensor are shown in Figure 1. Figure 1a shows gun types. There are a 92-type pistol and a 77-type pistol, and corresponding bullets, from top to bottom. In Figure 1a, the 92-type pistol weighs 0.76 kg, the trigger gravity is 2–3 kg, and the bullets used are 92-type pistol bullets. The 77-type pistol weighs 0.5 kg, it has a trigger attraction of 2–3 kg, and the bullets used are 64-type pistol bullets. In addition, Figure 1a also shows sensors and gloves. The sensors can be easily attached to the index finger of gloves, which can monitor the trigger action in real-time. Figure 1b shows the trigger-pulling actions of a tester wearing smart gloves for the two types of guns and an AD module. There are differences in trigger attraction between different gun types, and there are differences in the testers’ strength and trigger pulling speed, which all contribute to the accuracy of shooting. Figure 1c shows a tester wearing smart gloves to test the 92-type pistol, and the gloves are also equipped with Bluetooth response settings. The red circle in Figure 1c is an LED bulb to reflect the action process of the tester during shooting in real-time. A specific Bluetooth device is shown in Figure 1d, which shows the smart glove transmission system. When the tester pulls and releases the trigger, Bluetooth will respond in real-time and transmit the signal to the intelligent device. Figure 1d shows the intelligent shooting system. The data can be used for the intelligent shooting system signal. The signal can control the human–computer interaction system and it can also compose the database of personnel. This shows a wide application value.

**Figure 2:**
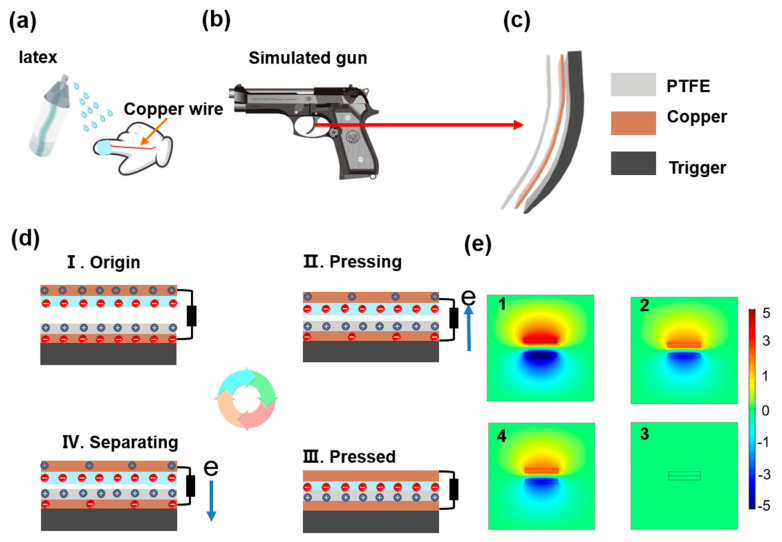
The manufacturing process and working mechanism. (**a**) Manufacturing process of SPTENG; (**b**) Attachment position of PTFE and simulation pistol; (**c**) Detail diagram of trigger; (**d**) Working mechanism of SPTENG; (**e**) COMSOL potential simulation diagram.

**Figure 3:**
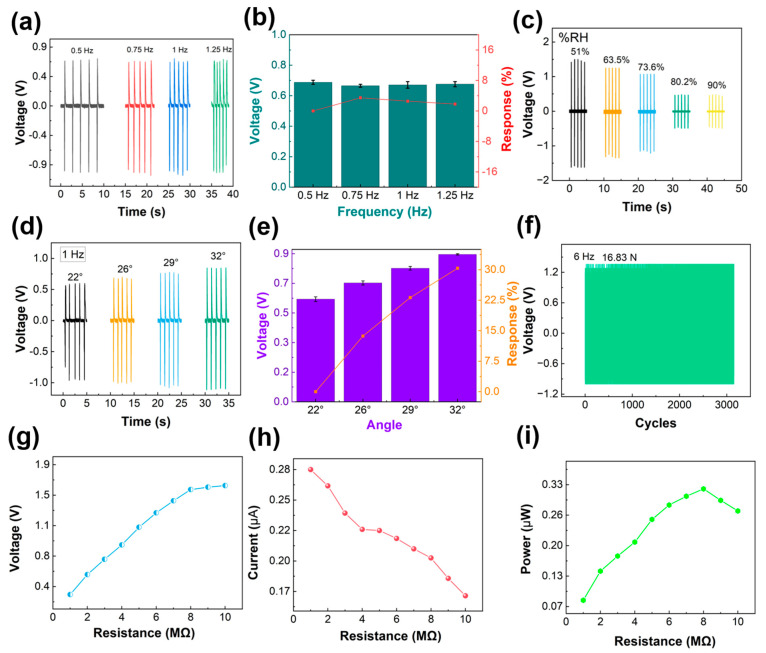
Electrical properties of SPTENG. (**a**) Voltage of SPTENG at different frequencies; (**b**) Response at different frequencies; (**c**) Voltage of SPTENG at different humidity; (**d**) Voltage of SPTENG at different angles; (**e**) Response at different angles; (**f**) Durability test; (**g**–**i**) The voltage, current, and power of SPTENG at different resistances.

**Figure 4:**
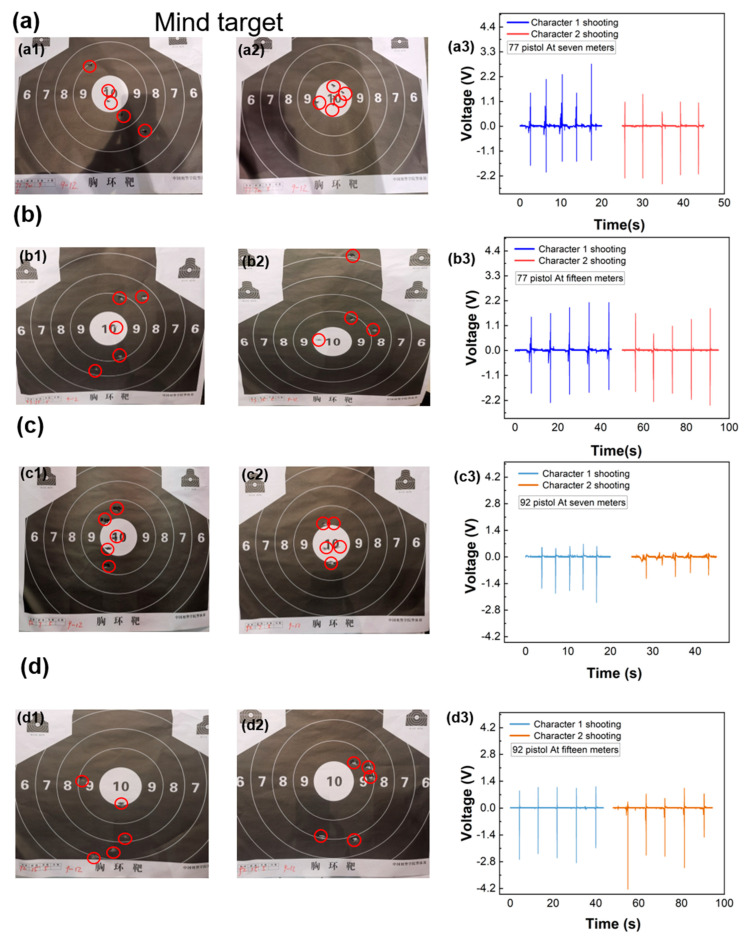
Actual test scene. (**a**: **a1**–**a3**) Two testers use a 77-type pistol to shoot target bitmap and shooting monitoring signal at 7 m; (**b**: **b1**–**b3**) Two testers use a 77-type pistol to shoot target bitmap and shooting monitoring signal at 15 m; (**c**: **c1**–**c3**) Two testers use a 92-type pistol to shoot target bitmap and shooting monitoring signal at 7 m; (**d**: **d1**–**d3**) Two testers use a 92-type pistol to shoot target bitmap and shooting monitoring signal at 15 m.

In the process of shooting, the convenient and wearable sensor device can not only collect information about the shooter’s action, but it also ensures the accuracy of the shooting action to the greatest extent. Therefore, latex is used as the positive electrode and PTFE as the negative electrode in this paper. As shown in Figure 2a, first of all the copper wire is placed on the finger, and then we sprayed latex on the finger surface with a sprinkling can. Because latex has high biocompatibility, there is no need to consider the problem of skin corrosion or damage. The latex was sprayed on the finger surface and then air dried naturally for 10 min. At this time, because the latex has shaping characteristics, it can wrap and fix the copper wire (Figure S1). After that, we cut PTFE into the shape of the trigger and then stuck it on the trigger, as shown in Figure 2b,c. Between the PTFE and the trigger, there is a copper foil with the same shape as the trigger for conducting electricity. Concerning Figure 2a, the trigger and finger together form a TENG, and this TENG has a high degree of freedom. Two electrodes can be contacted at different positions, and different signals can be monitored through contact at different positions. According to this characteristic, we can monitor the signal when the number of hit rings is high, which can be used for reference in monitoring shooting. Figure 2d shows the working mechanism of the SPTENG when shooting. When the finger is not in contact with the trigger, the SPTENG is in a non-contact state. Latex and PTFE have different electronegativities, so the potential is generated. The simulated potential is shown in Figure 2e-1. The potential distribution of the latex layer and the PTFE layer under open circuit conditions in four stages was calculated by COMSOL multi-physical simulation software. The charge density of the PTFE surface is set to 0.175 nC/cm^2^. The charge density of the latex surface is set to −0.18 nC/cm^2^. When the finger approaches the trigger, a current is generated in the load circuit due to electrostatic induction (Figure 2d-II) and the potential decreases continuously (Figure 2e-2). When the finger touches the trigger, the charge jumps. At this time, the charges of the two friction layers are balanced and no current is generated (Figure 2d-III). Meanwhile, the potentials of the two friction layers are balanced (Figure 2e-3). When the finger separates from the trigger gradually, due to friction electrification, the charges of the two friction layers are unbalanced and the potential gradually increases (Figure 2e-4), so an induced current will be generated in the load circuit (Figure 2d-IV). Therefore, the SPTENG can sense the tiny motion of shooting, which has great application value in the field of monitoring shooting.

Excellent electrical performance is an important index of TENG sensing equipment. In this paper, the electrical performance of the SPTENG is tested. It is tested by a stepping motor. The SPTENG required for the test is composed of materials of the same size as the finger and trigger. The latex size is 10 × 10 mm and the PTFE is 3 × 3 mm. Figure 3a shows the electrical characteristics at different frequencies. The frequency is controlled by step motor. The voltages are 0.688 V, 0.665 V, 0.671 V, and 0.676 V, respectively, when the frequencies are 0.5 Hz, 0.75 Hz, 1 Hz, and 1.25 Hz. This shows the output stability of the SPTENG at different frequencies. On the other hand, it can be seen from the figure that with the increase in test frequency, the number of peaks increases. For example, at 0.5 Hz, it takes 10 s to generate five peaks, while it takes 5 s to generate five peaks at 1 Hz. Therefore, the SPTENG has strong robustness under different motion frequencies. Figure 3b shows the response at different frequencies. When the frequency is 0.5 Hz, 0.75 Hz, 1 Hz, and 1.25 Hz, the response is 0%, 3.4%, 2.5%, and 1.8%, respectively. The response formula is as follows,

(1) $$R\%=\left|\frac{V_{0}-V_{i}}{V_{i}}\right|\times 100\%$$

where *V*_0_ and *V_i_* are the outputting voltage of first data and other voltages of angle. This also proves the stability of the SPTENG. Figure 3c shows the voltage signals of the SPTENG under different humidity conditions. Considering the wet environments that may be encountered during shooting, this paper tests different humidity environments. The test results show that the electrical signal decreases with the increase of humidity. When the humidity is 51%, 63.5%, 73.6%, 80.2%, and 90%, the corresponding voltages are 1.47 V, 1.25 V, 1.07 V, 0.47 V, and 0.43 V, respectively. This is because, with the increase in humidity, the effect of triboelectrification will be weakened. This test shows that the SPTENG can be applied in an environment with high humidity. Meanwhile, for testing the influence of sweat, the tester dipped the finger in water and then put on the latex glove to pull the trigger, and the voltage was still generated. The average voltage was 1.25 V. The relative humidity chart and the sweat simulation test voltage are shown in Figure S2. Figure 3d shows the voltage signals at different angles. With the increase in testing angle, the voltage also increases. When the angles are 22, 26, 29, and 32, the voltages are 0.59 V, 0.68 V, 0.77 V, and 0.85 V, respectively. Figure 3e shows the response at different angles. When the angles are 22, 26, 29, and 32, the responses are 0%, 13.68%, 23.14%, and 30.37%, respectively. This shows that there are different responses to different angles. Figure 3f shows the durability test of the SPTENG after 3400 cycles. The applied force was 16.83 N, which is shown in Figure S3. According to the figure, the voltage can be kept at 1.36 V after 3400 cycles. It shows that it can be carried out in the SPTENG for a long time. Figure 3g–i show the voltage, current, and power of the SPTENG at different resistances. As the resistance increases, the voltage increases also, and the current decreases. When the resistance is 8 MΩ, the power achieves its maximum. The maximum voltage, current, and power are 0.1.64 V, 0.28 μA, and 0.32 μW. Finally, we tested the relationship voltage with trigger gravity. When we pulled the trigger, the voltage was 0.82 V, and the trigger gravity was 26.43 N, which is shown in Figure S4.

The SPTENG can monitor the shooting movements of different people. Figure 4 shows the shooting results of two people in the same state. As shown in Figure 4a, Tester A and Tester B used a Type 77 pistol to shoot the target at 7 m. As shown in Figure 4(a1,a2), Tester A hit 8, 9, 9, 10, and 10 rings, respectively, and Tester B hit 10 rings five times. According to the target bitmap, Tester B is more stable than Tester A, and from the electrical signal (Figure 4(a3)), Tester A’s signal fluctuates greatly while Tester B’s signal is relatively stable. Therefore, in the shooting of this group of test subjects, B is relatively stable. However, in the test at 15 m distance, Tester A was more stable than Tester B, as shown in Figure 4b. Tester A hit 8, 8, 9, 9, and 10 rings, respectively, while Tester B hit 6, 8, 9, and 10 rings, respectively (the reason one ring is missing is that one shot missed the target). In Figure 4(b3) it is clearly seen that in the voltage of the two people shooting, Tester 1’s signal is more stable. Tester 2 missed the target the first time. This is because Tester 2 was not comfortable in long-distance shooting, resulting in the aim deviating from the target. Figure 4c,d show the shooting situation of two testers using 92-type pistols at different distances. In Figure 4c, their scores are 9, 9, 9, 10, and 10 rings, respectively. Although their signals are different from the electrical signals, their respective signals are stable. In Figure 4d, both rings of Testers 1 and 2 are unstable. Meanwhile, their voltage is unstable too. The relationship between voltage and rings of different people shooting with different guns at different distances is shown in Figure S5. Compared with different shooting distances, the testers used more time to aim and shoot in the 15 m shooting. For example, when the testers used the Type 77 pistol and shot targets at 7 m, Tester A took an average time of 3.83 s and Tester B took an average time of 4.71 s. However, when they shot at 15 m, Tester A took an average time of 8.98 s and Tester B took an average time of 8.78 s. It is seen that when they used the Type 92 pistol the same condition happened. This shows that, as the shooting distance increases, the shooting time also increases. Testers need more time to aim and adjust. Meanwhile, we tested different frequency shooting conditions. The data are shown in Figure S6. The tester shot six times total, three times slowly and three times quickly. It is obvious that when the tester shot slowly, the voltage was stable and can reflect the shooting frequency. When the tester shot quickly, however, the voltage was unstable and can also reflect the shooting frequency. It proves that different trigger frequency generates different voltage. With pulling the trigger faster, the voltage becomes unstable. Finally, we lit up an LED as an indicator, as is shown in Video S1. The SPTENG provides a special application for shooting.

**Figure 5:**
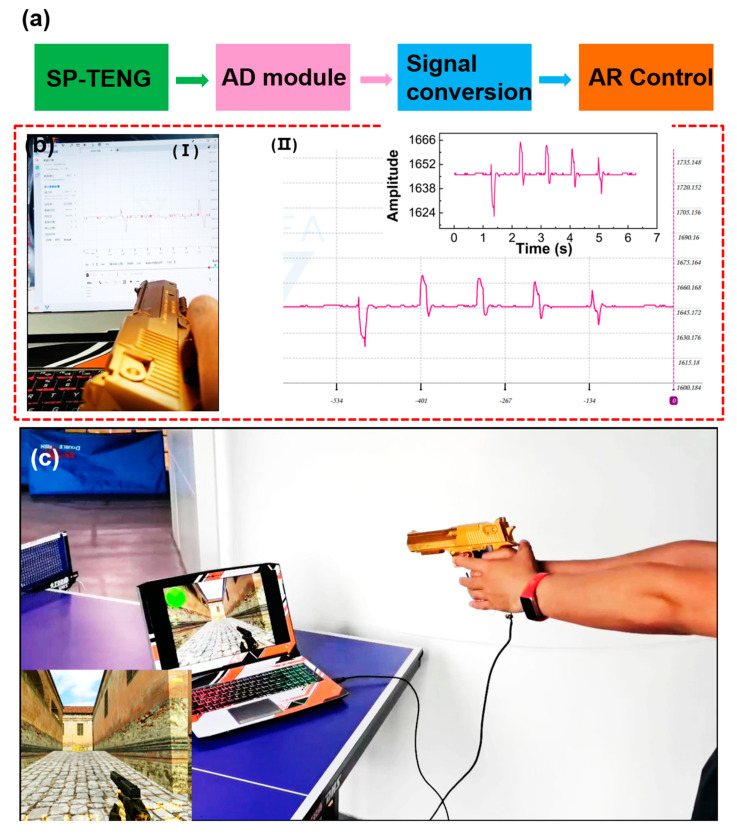
Human–computer interaction system. (**a**) Program diagram of human–computer interaction system; (**b**) Schematic diagram of signal transmission and detail drawing; (**c**) Scene diagram of human–computer interaction.

The Internet of Things, the application of 5G technology, and wearable devices can help the military and police better conduct virtual training. However, the existing equipment has the problems of large volume and high cost. TENG has the characteristics of low cost, convenience, and wearability. It can be used as a sensing unit for human–computer interaction. A TENG-based auxiliary equipment system for police man–machine interaction is shown in Figure 5a. It first converts the sensing signal through the AD module and converts the electrical signal into a digital signal, which is transmitted to the computer terminal, and, finally, the intelligent system recognizes the signal through machine learning. The system has the advantages of low cost, low delay, and high fault tolerance. It can be an excellent aid to police training. At the same time, it can enhance the immersion of users in the training process and enhance the entertainment of trainers in the use process. Figure 5b shows the signal transmission process. As shown in Figure 5b, when the trigger is pulled, the computer terminal displays a responsive sensing signal (Video S2). For signal processing, the peak search algorithm is mainly used to detect the extreme points whose amplitude is greater than a certain threshold and the distance is more than 0.1 s. The intelligent system intercepts the received signal sequence every second and detects the number of extreme points that meet the conditions as induction signals. Each induction signal is an active control in the virtual game, so the game terminal will respond to each trigger. For adding more sensing performance, multiple TENGs were added to the pistol. As in Video S3, there are three sensing units where are on the trigger, grip seat, and slide. The thumb of the left hand and the index finger and middle finger of the right-hand wear latex gloves, respectively. When the thumb of the left-hand pulls the slider, there is a sensing signal. When the index finger pulls the trigger, the middle finger will clench the grip seat. Meanwhile, there are signals being generated also. These will generate a touch map. Figure 5c shows the actual interaction scenario. When the tester pulls the trigger, the virtual game pulls the trigger at the same time, which is due to the induction signal generated at this time. In the test process, it can be seen that every time the tester shoots, the virtual character shoots at the same time, as in Video S4. Therefore, the human–computer interaction sensing system can realize the immersive shooting virtual scene, which provides a certain reference for intelligent military and police visual training equipment based on the Internet of Things.

## 4. Conclusions

The development of police equipment technology depends on the development of material technology, and the application of nanotechnology to police equipment, which is popular all over the world, has opened up a new space for the development of new material technology. This research is based on the new energy nanotechnology and is dedicated to improving the science and technology of police equipment, serving police skills training and actual combat, and focusing on the future development of wearable intelligent devices such as self-powered smart gloves, which may bring about significant technological changes in police equipment. In this paper, latex and PTFE are used to assemble a TENG which is used to monitor shooting and create a virtual interactive system. It can monitor the stability of shooting action. Using simple spraying technology to spray latex on fingers, because latex is biocompatible with the human body and is a positive electrode material, latex is used as the positive electrode. PTFE is attached to the trigger as a negative electrode. It can judge the stability of shooting. At the same time, a human-computer interaction system is used to collect and identify signals. As a sensing device for the human–computer interaction system, the SPTENG provides accurate sensing signals and connects real shooting action with a virtual game, providing an entertaining experience for the military police. Generally speaking, the SPTENG can provide a low-cost and universal solution for the stability of shooting action and human-computer interaction systems.

## Data Availability

The data presented in this study are available on request from the corresponding author.

## Supplementary Materials

The following supporting information can be downloaded at: https://www.mdpi.com/article/10.3390/ma15186228/s1, Figure S1: The latex attaches to the finger and PTFE on the trigger.; Figure S2: Different relative humidity test and voltage of sweat simulation.; Figure S3: The applied force of durability test.; Figure S4: The relationship of voltage with trigger gravity; Figure S5: Relationship between voltage and rings of different people shooting with different guns at different distances.; Figure S6: The voltage of different trigger frequency. Video S1: SPTENG lights LED; Video S2: The signal transmission; Video S3: Multi-point control; Video S4: The Man–machine interaction system.
