# Supplementary material for: A Self-Powered Triboelectric Nanogenerator Based on Intelligent Interactive System for Police Shooting Training Monitoring and Virtual Reality Interaction
Source: Materials (Basel). 2022 Sep 8;15(18):6228. doi: 10.3390/ma15186228 (PMC9500841; doi:10.3390/ma15186228)
Supplement: Supplementary file 1 [file materials-15-06228-s001.zip › materials-1877842-supplementary.pdf]

A Self-Powered Triboelectric Nanogenerator Based on Intelligent Interactive System for Police Shooting Training Monitoring and Virtual Reality Interaction

Songyang Li [1], Changjun Jia [2], Fengxin Sun [2] and Yongsheng Zhu [2,*]

[1] Police Skills and Tactics Training Department, Criminal Investigation Police University of China, Shenyang 110035, China
[2] Physical Education Department, Northeastern University, Shenyang 110819, China
* Correspondence: 2001276@stu.neu.edu.cn

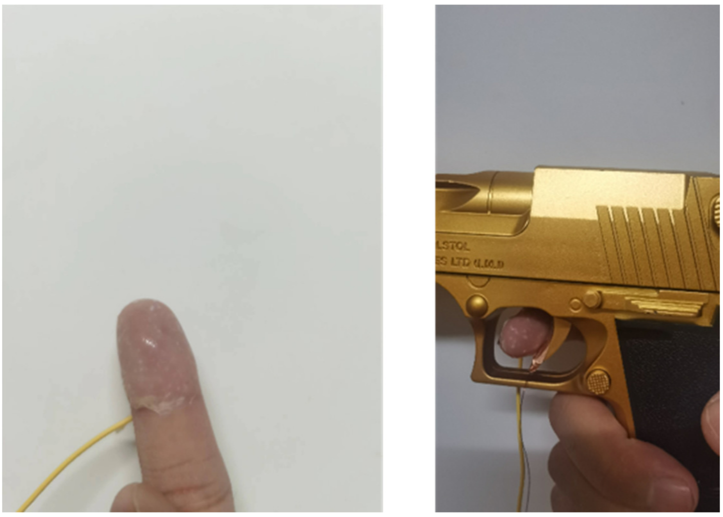

Figure S1. The latex attaches to the finger and PTFE on the trigger.

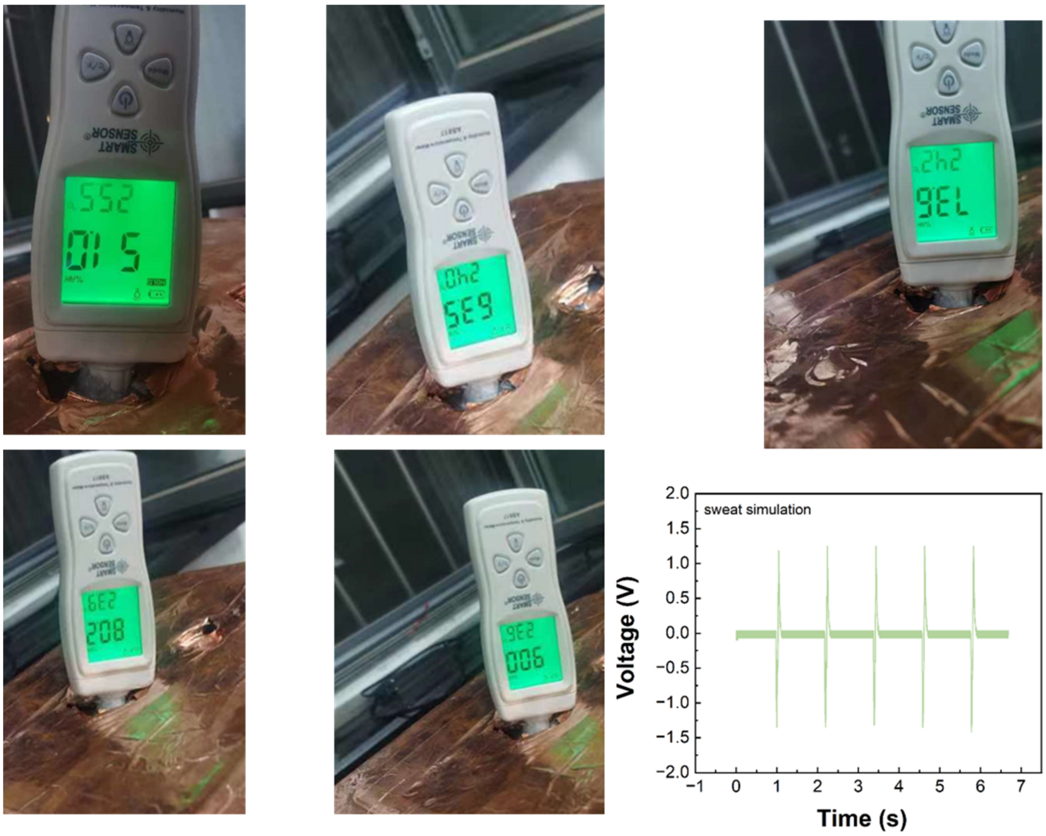

Figure S2. Different relative humidity test and voltage of sweat simulation.

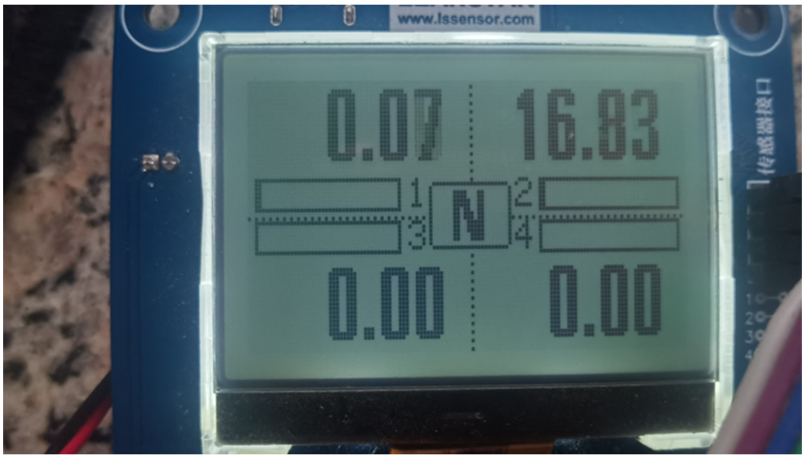

Figure S3. The applied force of durability test.

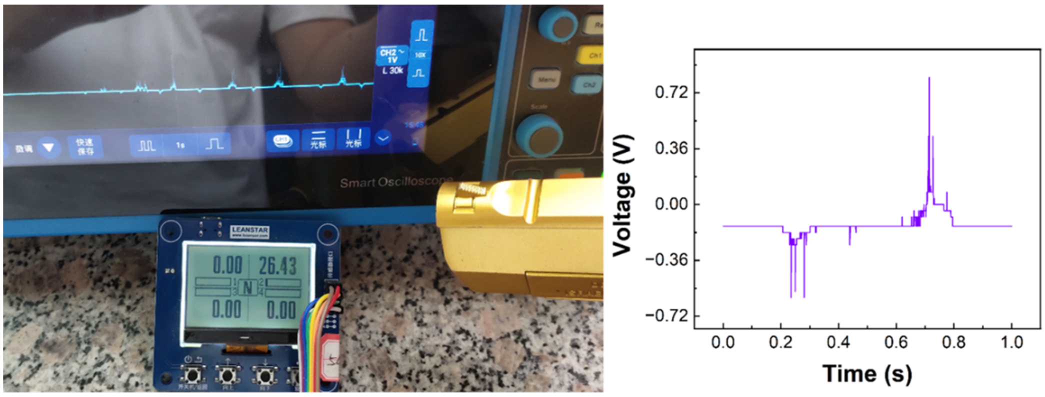

Figure S4. The relationship of voltage with trigger gravity.

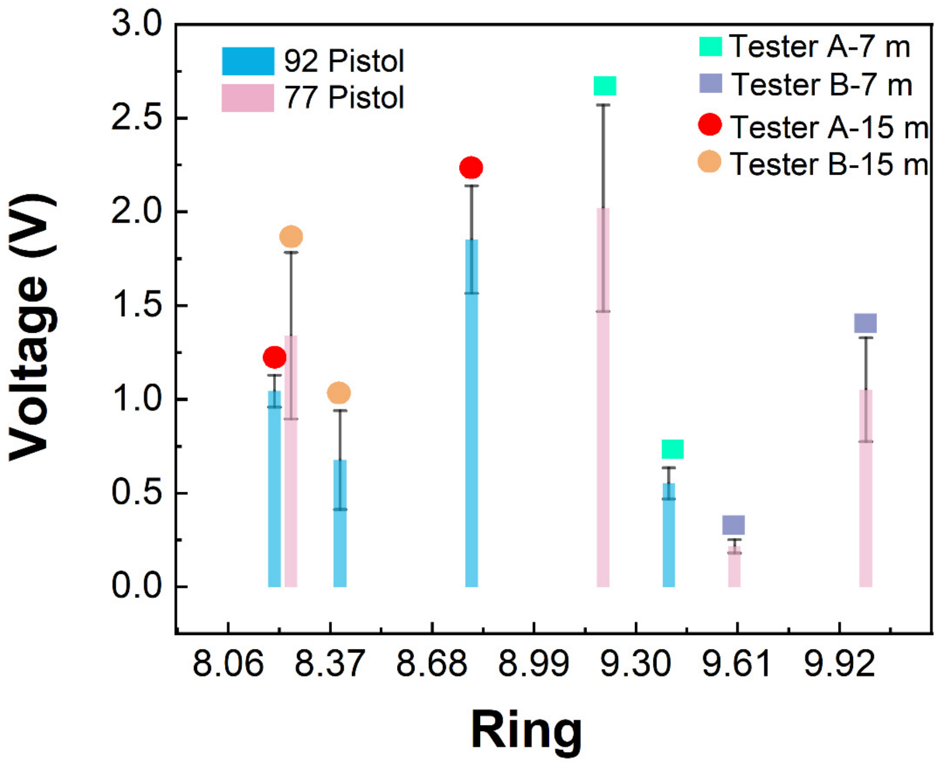

Figure S5. Relationship between voltage and rings of different people shooting with different guns at different distances.

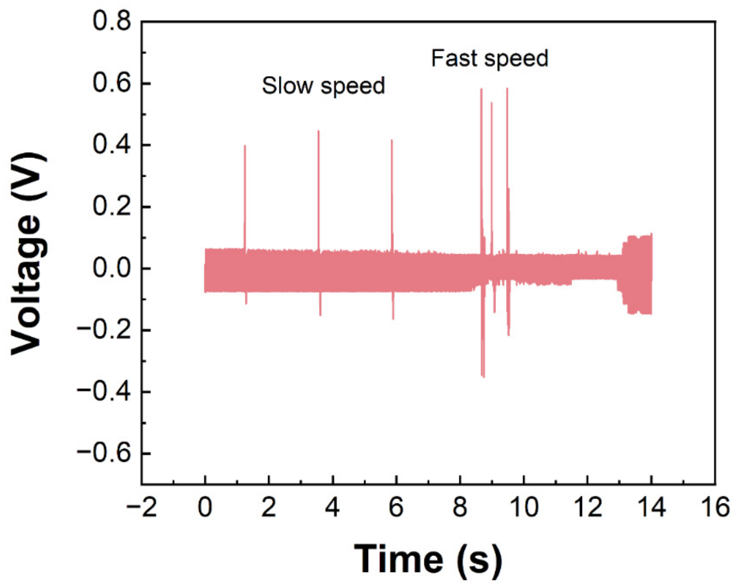

Figure S6. The voltage of different trigger frequency.

Movie S1: SPTENG Lights LED.
Movie S2: The signal transmission.
Movie S3: Multi-point control.
Movie S4: The Man-machine interaction system.
